# Genetic Rearrangements in Different Salivary Gland Tumors: A Systematic Review

**DOI:** 10.7759/cureus.61639

**Published:** 2024-06-04

**Authors:** Elham Albalawi

**Affiliations:** 1 Pathology, University of Tabuk, Tabuk, SAU

**Keywords:** ktn1-prkd1, myb-nf1b, ppp2r2a::prkd1, polymorphous adenocarcinoma, salivary duct carcinoma, adenoid cystic carcinoma, salivary gland tumors

## Abstract

Salivary gland tumors (SGT) encompass a wide range of neoplasms, each with its own unique histological type and clinical presentation. This review hones in on prevalent subtypes of SGTs, including adenoid cystic carcinoma (ACC), salivary duct carcinoma (SDC), and polymorphous adenocarcinoma (PAC). The articles, identified through specific keywords, were meticulously screened in databases like PubMed, Scopus, Google Scholar, and Web of Science from 2018 to 2023. Eight articles delved into genetic modifications among the selected SGT types. A fusion protein known as MYB-NF1B is typically associated with ACC, promoting cell proliferation while inhibiting apoptosis. The presence of MYB modifications in ACCs is a beacon of hope, as it is linked to a more favorable prognosis. In contrast, SDCs often exhibit HER2 expression.

The invasive nature of SGTs contributes to their resistance to treatment. In the case of PAC, the role of PRKD1 is particularly noteworthy. PRKD1, integrated with other genes from the PRKD1/2/3 cluster, helps to differentiate PAC from other diseases. Furthermore, the genetic profiles of KTN1-PRKD1) and PPP2R2A:PRKD1 are distinct. The significant genetic variability among SGTs necessitates meticulous examination. This field is in a constant state of evolution, with new discoveries reshaping our understanding. Genetics is a key player in deciphering SGTs and tailoring treatments. This complex neoplasm demands ongoing research to uncover all genetic influences, thereby enhancing diagnostic methodologies, therapeutic strategies, and patient outcomes.

## Introduction and background

Salivary gland tumors (SGTs) encompass a wide array of growths that can manifest in various areas within the oral and maxillofacial regions. These tubuloacinar exocrine organs begin their development during the sixth to eighth week of intrauterine life. The parotid gland, known to originate from the ectoderm embryonic layer, and the submandibular and sublingual glands, originating from the embryonic endoderm, are well-established [1, 2]. Salivary gland tumors can present with a diverse range of clinical characteristics, spanning from benign to malignant. Our understanding of SGTs has made significant strides, with a greater emphasis on genetics in research. Unraveling the genetic changes associated with different subtypes of SGT has unveiled new perspectives on their development, behavior, and treatment possibilities. It is widely acknowledged that specific genetic alterations can dictate the clinical trajectory of an SGT, with some alterations directly impacting therapeutic approaches. This new knowledge instills optimism for more efficient and minimally invasive treatments, indicating a significant transformation in the management of SGT.

The pathological classification system for SGT has recently experienced significant growth, with more than 30 different tumor types identified according to the latest World Health Organization (WHO) classification system released in 2017 [3-5]. Mucoepidermoid carcinomas (MEC), adenoid cystic carcinomas (ACC), and salivary duct carcinomas (SDC) are the most significant subcategories in the field of biological research. Mucoepidermoid carcinomas consist primarily of well- and moderately differentiated carcinomas that originate primarily from the parotid glands [6]. Another type of malignant tumor, low-grade polymorphous adenocarcinoma (PLGA), arises from minor salivary glands. It is more commonly found compared to ACC, surpassed only by pleomorphic adenoma and MEC, according to Darling et al. [7]. Anjali et al. [8] have identified a second variant, polymorphous adenocarcinoma (PAC), characterized by cytologic uniformity and architectural diversity.

Minor SGTs make up approximately 7%-11% of all tumors and account for approximately 19%-26% of all malignancies [7]. Despite advancements in diagnostic methods and the increased precision needed to categorize tumors into different forms of cancer [9], differentiating between diseases remains challenging. One such example is the relationship between intraductal papillary mucinous neoplasm (IPMN) and mucinous adenocarcinoma (MAC), where MAC is characterized by a recurring mutation of AKT1 p.E17K [10,11]. The identical genetic alteration can also be found in IPMN, which displays histopathological characteristics similar to those of mucinous adenocarcinoma [12]. The relationship between the two lesions remains a subject of debate. Intraductal papillary mucinous neoplasm can be considered a different MAC lesion, precursor, or subtype. Numerous genomic evaluations have identified characteristic gene modifications on chromosome 17 as distinctive to SDC. In contrast, ACC is marked by changes in chromosomes 6, 8, and X. Conversely, MEC initiation and development are linked with alterations occurring in chromosomes 9, 11, 15, and 19 [13, 14]. Moreover, there is an observed misregulation of genes located on chromosome 16 in the case of basal cell carcinoma. It has been established that carcinoma ex pleomorphic adenoma displays chromosomal imbalances, specifically with chromosomes 8 and 12. In the genetic landscape of PAC, changes have been noted in chromosomes 1, 2, 14, 19, and X [15].

Genetic alterations known as translocations have been detected in various types of malignant tumors and serve as crucial tools for diagnosis. Moreover, the comprehension of diagnostic worth and genomic alterations and their predictive and prognostic importance is gaining paramount relevance [16]. The article delves into the evolving landscape of genetic research in SGTs. Our goal is to provide a comprehensive overview of the role of genetics in understanding the pathogenesis, classification, diagnosis, and management of SGT. This review focuses explicitly on ACC, SDC, and PAC among salivary tumors, underscoring their significance in the field.

## Review

Methodology

Study Design and Strategies

The present research used a designated search approach, obtaining cohort or case study reports from databases such as PubMed, Web of Science, and Scopus, spanning the period of 2018 through June 2023. The keyword terms "adenoid cystic carcinoma" AND "genetic alterations" AND "fusion proteins"; "salivary duct carcinoma" AND "genetic alterations" AND "fusion proteins"; "polymorphous adenocarcinoma" AND "genetic alterations" AND "fusion proteins" were used for the search strategy. These specific terms were chosen to ensure a comprehensive search for studies focusing on the genetic alterations and fusion proteins in the specified types of carcinoma. The Preferred Reporting Items for Systematic Reviews and Meta-Analysis (PRISMA) protocol were followed for the study [17]. The papers incorporated in the investigation were those that delved into the somatic aberrations found in SDC, ACC, and PAC or cribriform adenocarcinoma of the salivary gland (CASG).

Study criteria

Inclusion Criteria

Only articles that specifically discussed the genetic alterations in SDC, ACC, and PAC were considered for this review. Clinical studies that reported on genetic rearrangements were also included. To maintain a clear focus, only articles published in English were considered. The decision to include an article was based on the relevance of its title and abstract to our investigation. If this information was insufficient, we retrieved and assessed the full-text paper. This stringent approach ensures that our study is based on the most relevant and reliable sources.

Exclusion Criteria

Articles published as conference abstracts, reviews, book chapters, letters to the editor, and personal opinions were excluded from the study. These types of articles were excluded as they often provide a summary or interpretation of existing research rather than presenting new data. Articles discussing screening procedures (like immunohistochemical findings) for SGT, whole genome sequencing, xenografts, and meta-analysis articles were also eliminated. Studies in languages other than English were excluded.

Data Extraction

Data extraction forms were meticulously employed to gather data from the selected studies. The results were disclosed in strict compliance with PRISMA protocols, ensuring the highest level of transparency and reliability in reporting the discoveries from this comprehensive examination. This commitment to protocol adherence underscores our dedication to rigorous and trustworthy research.

Results

Study Selection

**Figure 1:**
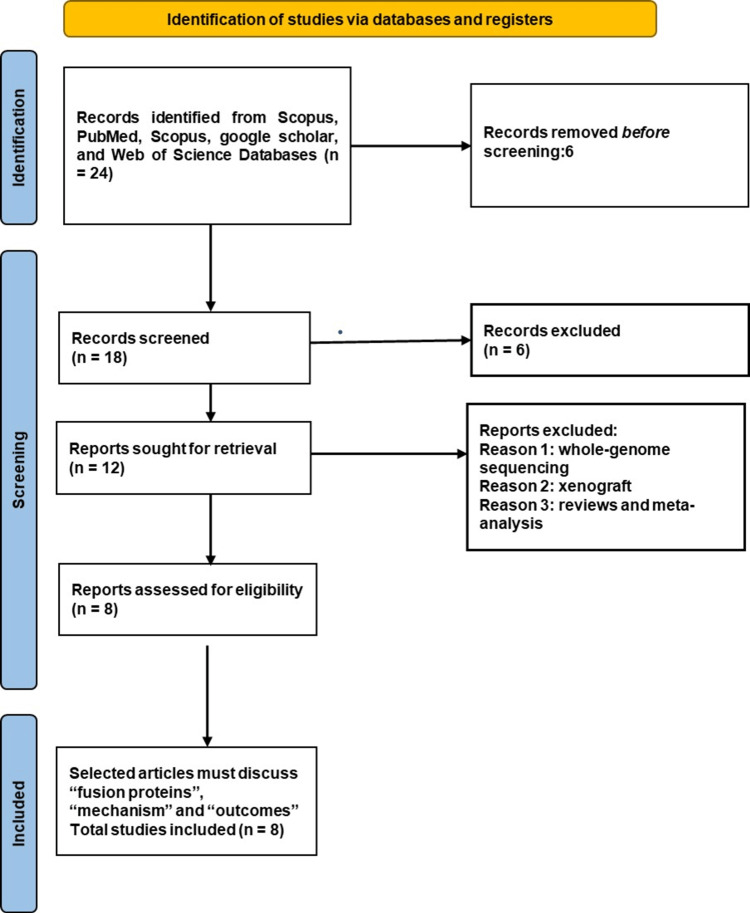
A PRISMA flowchart outlining the study selection process PRISMA: Preferred Reporting Items for Systematic Reviews and Meta-Analysis

Salivary gland tumors represent a wide range of neoplastic conditions; while many remain benign, many progress to malignancy. Distinctive histological subtypes are observed in malignant SGTs, each demonstrating unique clinicopathological characteristics and prognostic implications. In recent years, genomic research has played a crucial role in unraveling the fundamental processes that guide the genesis and progression of these tumor formations. This scholarly review delves into understanding how genetic variables impact specific types of SGT, emphasizing ACC, SDC, and PAC. Figure 1 illustrates the PRISMA flowchart. The initial search across PubMed, Scopus, Web of Science, and Google Scholar databases yielded 24 articles. After eliminating duplicates, six papers were excluded based on our criteria. Out of approximately 18 works reviewed, six were further excluded due to focusing on xenograft studies, reviews, meta-analyses, or whole-genome sequencing. Twelve papers remained for in-depth evaluation, with eight specifically addressing fusion proteins, their mechanisms, and resultant outcomes. These papers are of significant importance as they provide crucial insights into the genetic variables impacting SGT.

Genetic Alterations in ACC Salivary Tumors

Research in molecular biology has revealed that most ACC tumors are characterized by persistent chromosomal translocations, which initiate the activation of the MYB oncogene or its similar gene, MYBL1. In the current study, three articles were selected based on somatic mutations (Table 1) [18-20]. This leads to distinctive alterations in gene expression patterns [18]. Numerous translocations are found within the MYB or MYBL1 genes. These cause shortening and subsequent overexpression of these genes and their product molecules [21]. Therefore, proteins such as MYB or A-MYB, triggered by C-terminal truncations, can cause a unique tumor phenotype of ACC. This phenomenon is similar to how MYB proteins functionally affect other types of cells and malignancies [18, 21, 22].

**Table 1:** Articles on adenoid cystic carcinoma (ACC) with various genetic rearrangements

Reference	No of patients	Somatic mutations	Genetic rearrangement
Brayer et al., 2023 [18]	56	MYB: 83.1%, MYBL1: 12.5%	-
Humtsoe et al., 2022 [19]	10	MYB-NFIB: 44%	MYB-NFIB
Andersson et al., 2020 [20]	14	MYB-NFIB: >50%	NOTCH or FGF-IGF-PI3K pathways

Fusion of MYB-NFIB in ACC

A critical genetic modification in ACC primarily involves the amalgamation of the MYB gene and the NFIB gene. This was a discovery made by Lin and his team in 2023 [23]. The fusion is due to a chromosomal translocation, which can be observed as a standard occurrence within ACC, specifically t(6;9)(q22-23;p23-24). The combined MYB-NFIB gene plays an active role in the evolution and advancement of ACC by facilitating increased cell multiplication while simultaneously suppressing apoptosis. There is a strong correlation between the Myc factor and the depletion of myoepithelial cells in solid-type ACC. Clinically pathological classifications, adverse prognoses, and high-grade transformations have been established [19,24]. Furthermore, the tyrosine-protein kinase kit (C-kit) expression has been reported to range from 80% to 100% by Tang et al. [25]. This high prevalence was observed primarily among minor cases of salivary stages III-IV. Such findings significantly correlate this observation with an unfavorable prognosis [26]. The histological subtype of the ACC dictates the manifestation of the expression of the C-kit. C-kits' expression is predominantly observed in the luminal cell stratum of tubular and cribriform variants. This implies that myoepithelial cells might not produce a C-kit because abluminal cells do not show any staining [26]. This may correspond to the more severe clinical progression seen with solid ACC variants, as pointed out by certain studies [26].

In a study of 123 salivary carcinomas, Mitani and his team investigated the role of the MYB-NFIB fusion gene in ACC. In addition, they examined salivary gland tissues with primary and metastatic ACCs, non-ACC salivary carcinomas, and normal salivary gland tissue [27]. They found that out of 89 ACC incidents (72 primary ACC and 17 metastatic), 26 showed expression of the MYB-NFIB fusion transcript via reverse transcription polymerase chain reaction (RT-PCR), which was confirmed by fluorescence in situ hybridization (FISH) research methods. This gene fusion expression was not detected in a single group of 34 non-ACC carcinomas [27]. The ability of normal salivary gland cells to form colonies and organoids seems to be enhanced by the presence of MYB-NFIB [28].

Activation of MYB in ACC

The research by Gao et al. [28] is significant as it reveals an elevated expression level of several genes associated with high MYB activity within ACC cells. Notably, they found significant increases in the expressions of ART3, EPHA7, SERPINE2, and SCRG1 [28]. This MYB activation is not limited to ACC cells, as other recognized targets for this gene, such as BIRC3, CDC2, and CXCR4, also show increased expression levels among MCF10A cells containing unchanged versions of both MYB and its fusion partner NFIB [20]. The study also identified potential pathways that could enhance the effects of MYB in ACC cases, with approximately 81% of the binding sites for TP63 within ACCs also bound by this oncogene. The researchers concluded that the interaction between NOTCH-MYB-TP63 was highly dependent on specific cell types, primarily within luminal cells (for NOTCH) and myoepithelial cells (for TP63), respectively (Table 1) [20,29].

Genetic Alterations in SDC Salivary Tumors

Typically affecting the parotid gland, SDC is an aggressive tumor in the salivary glands [30]. The idiosyncratic challenges that SDC poses in terms of detection and management underscore the importance of demystifying its genetic components. From a clinical perspective, SDC follows an aggressive trajectory, with patients typically experiencing distant and regional metastases. Tragically, within a period of three to four years after diagnosis, many patients succumb to this disease [31].

Amplification of HER2 in SDC

Salivary gland tumors have a wide range in terms of HER2 positivity, fluctuating between 0% and 43%. Salivary duct carcinomas exhibit the highest frequency, showing genomic and morphological characteristics similar to invasive ductal carcinomas found in breast tissues [32]. Research on the pathogenesis of SDC has revealed that more than a third of SDC patients reflect an overexpression of HER2, paralleling its breast equivalent [31]. While clinical evaluations exploring HER2-targeted treatments using trastuzumab or lapatinib without concurrent chemotherapy have not produced substantial clinical advantages for SGC, there is hope [33]. A Japanese research study that combined docetaxel and trastuzumab therapies reported an overall response rate of around 70% for those suffering from IHC3+ or FISH-confirmed HER2-positive SDC [34].

PIK3CA Mutations in SDC

Salivary duct carcinoma is a severe cancer type that often manifests itself in later stages. Variations or increased copies of the gene responsible for producing the p110a catalytic subunit of PIK3CA p110a, along with diminished levels of the PTEN, are recognized as triggers for activating the PI3K pathway, thus indicating potential therapeutic targets [35]. A comprehensive study involving numerous patients with various types of tumors revealed that only this PIK3CA p.H1047R mutation could independently predict a positive response to inhibitors targeting the PI3K pathway [36]. Moreover, activation of this signaling pathway may occur due to deletion or loss of function from PTEN, an element that negatively regulates activity within the same route, further complicating matters. Ettl et al. discovered the appearance of PTEN deletion in 16 of 24 SDC cases using FISH [36]. In several genomic studies, somatic mutations related to SDC have been detected. These include gene alterations such as EGFR (20%), PDGFRA (27%), HRAS (27%), KIT (33%), PIK3CA (53%) and PTEN (53%). This revelation has dramatically expanded the possibilities for future targeted treatments [37].

HNRNPH3-ALK Fusion in SDC

Based on the somatic mutations, only one study was considered in the current study (Table 2) [38]. An ALK gene rearrangement was found for the first time in a de novo cancer. This reconstructed gene resulted from the fusion of 3'ALK on chromosome 2 to 5'UTR of the HNRNPH3, which is prevalently expressed throughout human cells and situated on chromosome 10. Dogan et al. [38] showed that this process resulted in the generation of a putatively functional frame product with the nomenclature HNRNPH3-ALK. The same study also reported that there were two instances of ALK fusions within SDC, whereby numerous ALK gene fusion partners had been previously observed in diverse cancer types. An example includes the EML4-ALK fusion found within lung carcinomas [39]. The regulation process for the expression of 3'ALK is guided by elements inherent within the HNRNPH3 promoter, while intriguingly, no HNRNPH3 coding exons are included in this unique fusion transcript. Providing further support for this novel genomic occurrence is evidence suggesting that tumor cells exhibit an actively translated fusion protein; this conclusion is supported via positive immunohistochemical staining with monoclonal antibodies specifically targeting ALK's C-terminal section of the tyrosine kinase domain [40].

**Table 2:** Article on salivary duct carcinoma (SDC) with genetic rearrangements

Reference	Period	No of patients	Somatic mutations	Genetic rearrangement
Dogan et al., 2019 [38]	December 1999 to July 2017	14	TP53: 100%, HER2: 50%	PIK3CA/HRAS, HNRNPH3-ALK

Genetic Alterations in PAC Salivary Tumors

Polymorphous adenocarcinoma and cribriform adenocarcinoma of the minor salivary gland (CAMSG) are neoplasms developed in the salivary gland, exhibiting similar morphological characteristics. These tumors, with their unique genetic alterations, are of significant importance in the fields of oncology and genetics. A substantial majority, about 95%, of PAC impacts the minor salivary glands in the upper parts of the aerodigestive tract. The palate is most often affected, accounting for around 60% of cases within a range spanning from 49% to as high as 87%. Based on our selection criteria, four articles were selected in the PAC category of SGT and are represented in Table 3 [41-44]. The disease manifests in various age groups, ranging from 16-year-old adolescents to individuals nearing their centennial years [41,45]. Alternative possible origins include the base of the tongue, inner cheek lining or buccal mucosa, mouth floor region, lip area, lateral surface of the tongue, and retromolar trigone area. It may also originate at the location of the sinonasal tract, and regions such as the oropharynx and nasopharynx can also be potential sites [45-47].

**Table 3:** Articles on polymorphous adenocarcinoma (PAC) with genetic rearrangements CASG: cribriform adenocarcinoma of the salivary gland

Reference	Period	No of patients	Somatic mutations	Genetic rearrangement
Sebastiao et al., 2020 [41]	-	37	PAC: 70%, CASGs: 80%	PRKD1 E710D: 56%; PRKD1/2/3- 13%
Jassim et al., 2020 [42]	-	1	PAC:100%	KTN1-PRKD1
de Jager et al., 2023 [43]	-	1	CASG: 100%	PPP2R2A-PRKD1
Xu et al., 2020 [44]	1993-2016	48	PAC: 38%	PRKD1- 73%; PRKD1, PRKD2, or PRKD3: 7%

PRKD1 Rearrangements in PAC

In the process of differentiating between various types of PAC, genetic rearrangements can play a significant role. It is found that more than 70% of the PAC cases exhibit mutations in PRKD1, and it is evident that PRKD1 variations are characteristic of this entity [48]. An example of a single-nucleotide mutation that affects a highly conserved amino acid in the kinase's catalytic loop is E710D. Since this modification boosts kinase activity and cell proliferation, it probably encourages PAC progression [49]. The PRKD1 gene translates into serine/threonine protein kinase D1; this plays an influential function across several signal transduction pathways associated with cellular adhesion, migration, vesicle conveyance, and survival factors [50]. Alongside PRKD1 are two other protein kinase D gene family constituents: PRKD2 and PRKD3 [51]. However, compared to conventional PAC scenarios where rearrangements rather than mutations occur in approximately four-fifths of CAMSGs, a classical form of PAC, they involve genes from the entire range, i.e., from PRDKD1 to PDRK3. These rearrangements give rise to common ARIDIA-PRKDI and DDX3X-PRKDI fusion events at the gene level, but their exact molecular implications remain undetermined [45]. A significant proportion (73%-89%) of PACs carry the PRKD1 E710D hotspot mutation, while a smaller fraction (6%-11%) possess fusions involving the PRKD1, PRKD2, or PRKD3 genes [44, 49, 52].

Fusion of KTN1-PRKD1 in PAC

Jassim and his team [42] discovered a unique fusion protein composed of KTN1 exon 9 and PRKD1 exon 12 (Table 3). Following whole exome sequencing (WES) and transcriptome sequencing (RNAseq), they detected an unprecedented intrachromosomal gene fusion within the tumor of a 39-year-old male patient, specifically in the parotid gland, kinectin1-PRKD1, which is encoded in KTN1 located in chromosome region 14q22. This particular gene, KTN1, codes for an essential member of the kinectin protein group incorporated into the membrane composition [42]. The principal function attributed to it involves serving as a receptor for kinesin. This motor protein plays a critical role in the motility of the vesicles, driven by kinesin itself. Furthermore, kinectins have also been observed to concentrate within integrin-based adhesion complexes (IACs) [53,54].

Previous studies have indicated that ARID1A-PRKD1 and DDX3X-PRKD1 fusions correlate with PRKD1 overexpression. This assertion also applies to the KTN1-PRKD1 fusion, which potentially triggers an analogous effect of amplified PRDKD expression. This hypothesis aligns with evidence of a gain-of-function point mutation (p.E710D) in PRKD1, documented in three-quarters of PAC cases [42,49].

PPP2R2A-PRKD1 Fusion in PAC

A recent investigation led by de Jager et al. [43] focused on a 58-year-old patient exhibiting the morphological characteristics of CASG (Table 3). The team discovered an unprecedented expression of the fusion transcript, consisting of PPP2R2A (exon 2) and PRKD1 (exon 11). This finding solidified the diagnosis of PAC. In this new arrangement between PPP2R2A: PRKD1, it is essential to note that the protein PPP2R2A acts as a regulatory subunit for the enzyme PP2A, one of the four primary serine/threonine phosphatases that play a critical role in negatively regulating cell growth and differentiation processes [55]. Previous reports have identified PPP2R22 as a fusion partner with CHEK1 and PLAG1 in conditions such as mature intrathoracic teratoma and lipoblastoma, respectively [56,57]. The unique rearrangement observed in lipoblastoma was when the entire PLAG coding sequence came under transcriptional control due to PPP2R2A::PLAG reorganization [43].

Discussion

Adenoid cystic carcinoma, a prevalent malignancy found primarily in major and minor salivary glands, is one of the most frequently diagnosed forms of salivary gland cancer. However, instances of ACC are also seen in other organs, albeit less frequently, and exhibit a wide range of clinical behaviors [58]. Adenoid cystic carcinoma is identified as an infrequent yet malignant SGT. It is recognized for its slow proliferation rate but is also noted for its tendency toward local infiltration and high chances of recurrence. Solid tumors denote the highest grade within the ACC classification system, which is directly correlated with an unfavorable prognosis, specifically in advanced stages leading to the development of distant metastases. The tumor cells are diminutive cuboidal structures that exhibit deep basophilic qualities with minimal cytoplasm [59]. The distinctive histological characteristics of this disease present substantial challenges for pathologists, underscoring the need for genetic information to understand its pathogenesis process better.

Numerous resistance mechanisms have been suggested for HER2-targeted therapy. These include the absence of the extracellular trastuzumab binding domain on HER2 receptors, increased levels of other tyrosine kinase receptors, or modifications in downstream components that lead to irregular PI3K/Akt/mTOR pathways [60]. The manifestation of HER2 in SGC exhibits high variability between and within various histological classifications. Among the 3,372 patients with 16 distinct subtypes of SGC, the incidence rate of HER2 positivity fluctuated from non-existent to as high as 43%. Predominantly observed in SDC and certain tumor subclasses originating from exocrine cells, a comprehensive meta-analysis study reported almost no evidence of HER2 expression [61]. From a clinical perspective, the PIK3CA mutations found in some SDC cases make it an attractive candidate for therapy. In two distinct cases of SDC, mutations in PIK3CA p.H1047R were observed [35]. Despite the potential benefits of inhibition of ALK in patients with SDC with rearranged HNRNPH3-ALK tumors, established knowledge of lung adenocarcinoma, a cancer with a similar genetic makeup, may guide treatment decisions in SDC with genetically similar tumors [38].

Studies have reported an annual incidence rate of PAC prevalence of roughly 0.051 cases per every set group of 100,000 individuals [46]. The presence of alterations within the same gene family in both PACs and CAMSGs, albeit through distinct mechanisms, namely a PRKD1 mutation or fusion with one of the three PRDK genes, suggests an intertwined molecular pathogenesis between these two entities [41]. This interconnectedness of PACs and CAMSGs at the molecular level adds another layer of complexity to our understanding of these diseases.

Instances of combined PAC and CAMSG characteristics could show either form of molecular alterations, as documented by Seethala [62] and Skálová et al. [63]. Molecular disparities have also been observed. Mutation in PRKD1 typifies PACs, while translocation in PRKD1-3 is a distinctive feature of CAMSGs. However, when the characteristics of both PAC and CAMSG overlap, molecular intersectionality is present. However, it has been revealed that mutation E710D in PRKD1 results in substantial augmentation of kinase activity within experimental models, which further leads to escalated indices for cell proliferation in vitro along with modifications to glandular structure within three-dimensional models using non-malignant breast epithelial cells [49]. Taking into account the high sequence identity shared among the kinase domain between PRKD1, PRKD2, and PRDK3, together with some subsets of PACs that exhibit an absence of somatic mutations at the PRKD1 hotspot or rearrangements within the gene family such as those found in low-grade PACs also known as PLGAs, it was hypothesized that alternative oncogenic mechanisms to mutation E710D in PDRK1 may influence other members of the gene family, thus playing a role in pathogenesis related to PAC, according to Piscuoglio et al. [64].

A study by Sebastiao and his team [41] discovered that most tumors in the histological spectrum of PAC are subject to genetic deviations that affect PRKD genes, accounting for a significant 78.4%. The researchers found a higher numerical appearance of PRDK1:E710D hotspot mutations in classical PAC cases, an increased frequency of rearrangements involving PRKD1/2/3 genes in CAMSG, and tumors exhibiting predominantly papillary patterns. These findings suggest that in SGT locations, rearrangements involving the PRKD/PRKD2 and PRKD3 genes are related to the diagnosis of cribriform adenocarcinoma, a tumor subtype previously connected to the base of the tongue, and a high risk of nodal metastasis [65]. A strong relationship between PRKD1 E710D somatic hotspot mutations and rearrangements within the same gene series was noticed, as they do not coexist. These discoveries lend acceptance to the theory suggesting that the diverse histologic spectrum of polymorphous adenocarcinomas could potentially stem from genetic alterations that play havoc with PRKD genes [66].

Interestingly, tumors lacking this particular fusion showed increased PLAG gene RNA expression compared to those with intact gene structure [57]. The latest discovery was an intact fusion between the promoter region and the starting site for transcription of the PPP22RA gene with PRDKD1 exon 11. This resulted in the formation of a fused product labeled PP222RA: PRDKD. This hybrid molecule contained a kinase domain that seemed truncated but was still expressed under the influence of the parent molecule, i.e., PP222RA. Consequently, de Jager and colleagues [43] speculated that the genetic rearrangement of PPP2R2A: PRKD1 could incite excessive expression of an operational PRKD1 kinase domain, thus facilitating the identification of CAMSG.

Limitations of the study

Salivary gland tumors are noted for their extreme diversity in histological classifications, clinical behaviors, and genetic modifications. Encapsulating all genetic elements that impact these tumors in a single scholarly article is strenuous, given that distinct subtypes may possess unique genetic profiles and clinical consequences. Specific genetic alterations within SGTs lack comprehensive characterization or adequate clinical data, obstructing the ability to draw solid conclusions. This obstacle can impair our understanding of the complete range of genetics that play a role in these tumors. It's clear that more research is needed to fully comprehend the genetic landscape of SGTs and its implications for clinical practice. While the article mentions potential targeted therapies based on genetics in the original article, it may need to be more detailed regarding practical challenges or limitations when administering these treatments. The effectiveness and availability of such interventions can vary greatly; therefore, not all patients will experience equal benefits. Therefore, our publication may not comprehensively discuss the application of genetic discoveries to clinical procedures or determine to what extent these breakthroughs have revolutionized the diagnosis, therapy, or prognosis of individuals with SGT.

## Conclusions

Salivary gland tumors exhibit a wide variety of genetic deviations, with each type of neoplasm having a distinct genomic profile that influences tumor growth and prognosis. This includes slow-growing ACC, aggressive SDC, and benign PAC. Fusion of the MYB-NFIB gene in ACC contributes to cell proliferation. It impedes programmed cell death, with ACCs demonstrating MYB alterations linked to improved results versus those with other gene changes. These genetic variations also provide avenues for potential targeted treatments. In the case of SDC, HER2 amplification, a notable genomic alteration, enhances cancer aggression and promotes uncontrolled cell growth. This opens the door for targeted therapies with promising clinical trial results, instilling hope for improved treatment options. Polymorphous adenocarcinoma is characterized by the PRKD1-E710D fusion gene, resulting from the rearrangement of the PRKD1 gene, yet it does not significantly affect tumor prognosis. These insights underscore the varied genetic influences on different types of SGT and emphasize the importance of specific genomic elements in their development and clinical progression. Research into the genomic traits of SGTs is rapidly advancing, continuously enhancing our understanding of genetic influences on tumor growth. This knowledge is vital for all, potentially revolutionizing these neoplasms' identification, management, and prognosis. Comprehending these genetic elements is critical to understanding tumor progression and therapeutic strategies. This document highlights crucial genetic changes associated with specific tumors, providing diagnostic markers and possible treatment paths. Continuous research is essential in the ever-evolving field of genetic modifications on SGTs. We aim to improve our understanding of these genetic changes, aiding in diagnostic precision, treatment plans, and prognosis, and ultimately improving patient quality of life.
